# The Nociceptive and Inflammatory Responses Induced by the Ehrlich Solid Tumor Are Changed in Mice Healed of *Plasmodium berghei* Strain ANKA Infection after Chloroquine Treatment

**DOI:** 10.1155/2024/3771926

**Published:** 2024-05-14

**Authors:** Maria de Fatima Rodrigues Aguiar, Meiriane Mendes Guterres, Eduarda Magalhães Benarrosh, Waldiceu Aparecido Verri, Cássia Calixto-Campos, Quintino Moura Dias

**Affiliations:** ^1^Laboratory of Neuro and Immunopharmacology (NIMFAR)-Oswaldo Cruz Foundation, Fiocruz Rondônia, Rua da Beira, 7671, BR 364, Km 3.5, Bairro Lagoa, Porto Velho, Rondônia, Brazil; ^2^Postgraduate Program in Experimental Biology (PGBIOEXP), Federal University of Rondônia, Campus-BR 364, Km 9.5, Porto Velho, Rondônia, Brazil; ^3^Department of Pathology, Laboratory of Pain, Inflammation, Neuropathy and Cancer, Center of Biological Sciences, State University of Londrina, Londrina, Paraná, Brazil; ^4^National Institute of Science and Technology on Neuroimmunomodulation (INCT-NIM), Oswaldo Cruz Institute, Oswaldo Cruz Foundation, Rio de Janeiro, Brazil; ^5^São Lucas University Center - São Lucas PVH, Porto Velho, Rondônia, Brazil

## Abstract

Comorbidities that involve infectious and noninfectious diseases, such as malaria and cancer, have been described. Cancer and malaria induce changes in the nociceptive and inflammatory responses through similar pathophysiological mechanisms. However, it is unclear whether malaria and antimalarial treatment can change the inflammatory and nociceptive responses induced by solid cancer. Therefore, the present study experimentally evaluated the effect of infection by *Plasmodium berghei* strain ANKA and chloroquine treatment on the nociceptive and inflammatory responses induced by the solid Ehrlich tumor in male BALB/c mice. On the 1^st^ experimental day, mice were infected with *Plasmodium berghei* and injected with tumor cells in the left hind paw. From the 7^th^ to the 9^th^ experimental day, mice were treated daily with chloroquine. The parasitemia was evaluated on the 7^th^ and 10^th^ days after infection. On the 11^th^ experimental day, mice were evaluated on the von Frey filament test, the hot plate test, and the paw volume test. At the end of the experimental tests on the 11th day, the peripheral blood of all mice was collected for dosing of IL-1*β* and TNF-*α*. The blood parasitemia significantly increased from the 7^th^ to the 10^th^ day. The chloroquine treatment significantly decreased the parasitemia on the 10^th^ day. The presence of the tumor did not significantly change the parasitemia on the 7^th^ and 10^th^ days in mice treated and nontreated with chloroquine. On the 11^th^ day, the mechanical and thermal nociceptive responses significantly increased in mice with tumors. The treatment with antimalarial significantly reduced the mechanical nociceptive response induced by tumors. The hyperalgesia induced by tumors did not change with malaria. The mechanical and thermal hyperalgesia induced by the tumor was significantly reduced in mice treated and healed from malaria. On the 11^th^ day, the volume of the paw injected by the tumor was significantly increased. The mice treated with chloroquine, infected with malaria, or healed of malaria showed reduced paw edema induced by the tumor. Mice with tumors did not show a change in IL-*β* and TNF-*α* serum levels. Mice with tumors showed a significant increase in serum levels of IL-1*β* but not TNF-*α* when treated with chloroquine, infected with malaria, or healed of malaria. In conclusion, the results show that malaria infection and chloroquine treatment can influence, in synergic form, the nociceptive and inflammatory responses induced by the solid tumor. Moreover, the mechanical antinociception, the thermal hyperalgesia, and the antiedema effect observed in mice treated with chloroquine and healed from malaria can be related to the increase in the serum level of IL-1*β*.

## 1. Introduction

Cancer is a chronic degenerative disease characterized by abnormal cellular proliferation and growth, responsible for increasing mortality worldwide [1]. Breast cancer is frequently manifested among women, especially in underdeveloped countries, which is a significant cause of death [1, 2]. Malaria is an infectious disease caused by a single-celled protozoan of the genus *Plasmodium* transmitted to humans through the bite of an infected female *Anopheles* mosquito [3–5]. The accumulated evidence shows that malaria and cancer could influence each other biologically given their evolutionary history and epidemiology [6]. In addition to the inflammatory response, malaria and cancer share clinical manifestations, such as pain. However, the interrelation between malaria and solid cancer, such as breast cancer, on the development of pain is not yet fully understood.

Pain and inflammation were the most devastating manifestations associated with cancer progress, negatively impacting the quality of life and causing disability in these patients [7, 8]. Cancer pain is a symptom often informed by patients since its diagnosis [9], and its intensity increases proportionally with the survival time of these patients [10, 11]. The incidence of cancer pain increases from diagnosis to the advanced stage [2], where patients with advanced cancer experience more intense pain than those in the initial stage of the disease [12]. Pain and inflammation induced by solid cancer can be evaluated using an undifferentiated carcinoma, such as the Ehrlich tumor, grafted to the paw or calf of mice [13, 14].

Pain is a common symptom of malaria. Studies show that headache, myalgia, abdominal pain, and joint pain are algic symptoms during malaria infection [15, 16]. The pathophysiological mechanism of pain and other symptoms can involve the release of inflammatory cytokines, especially TNF-*α*, IL-1, IL-6, IFN-*γ*, IL-8, IL-10, and IL-13 [17–21], during the inflammatory response induced by malaria. IFN-*γ*, IL-2, IL-5, IL-6, and IL-12 were increased in mild malaria, whereas TGF-*β*, TNF, IL-10, and IL-1*β* were particularly elevated in cerebral malaria [22]. Evidence also shows that malaria can also induce hypoalgesia, as observed in some experimental models of pain [23]. Mice infected with *Plasmodium berghei* presented a minor nociceptive response to noxious chemical, mechanical, and thermal stimuli, and these effects were directly associated with increased parasitemia [23]. In developing countries, malaria can coexist with cancer, but the consequences of the interaction between these diseases are not fully understood [6].

Evidence suggests that malaria and tumors share pathophysiological events, such as stimulation of innate and adaptive immune responses, especially the inflammatory response [24, 25], generating competition and mutual influence between both diseases [26]. Data show that the incidence of malaria is inversely proportional to the incidence and mortality of several types of cancer [27]. Mice infected with *Plasmodium berghei* and transplanted with leukemic cells survived more time compared to mice inoculated with only leukemic cells [26]. Furthermore, mice with malaria and tumors presented a minor mass of lymphomatous tissues compared to animals that received only the tumor, indicating the inhibitory effect of malaria on Lewis tumor growth [26].

Chickens infected with avian malaria did not show significant growth of chicken tumor I [28]. *Plasmodium* infection suppresses the growth of tumor and metastasis through activation of the innate and adaptive immune systems [24]. Malaria infection increases the level of TNF-*α* and interferon-gamma (INF-*γ*), the activation of natural killer cells, the proliferation of tumor-specific T cells, and the activity of CD8^+^ T cells in mice with Lewis lung cancer [24]. *Plasmodium* infection inhibits the growth of 4 T1 tumor cells and increases the survival of tumor-bearing mice [29]. This inhibitory effect on tumor growth is associated with the induction of antitumor immune responses that are mediated by CD8^+^ T cells. Evidence indicates that malaria has antitumor activity.

On the other hand, evidence also suggests that malaria can favor the development of several types of cancer [27, 30–32]. Cases of Burkitt lymphoma, an aggressive non-Hodgkin lymphoma, have a high prevalence in areas with stable malaria transmission [33]. The cohort study indicates an association between confirmed malaria cases in individuals of endemic origin and cases of lymphoid neoplasm [34]. The capacity of malaria to promote the development and evolution of cancer can be associated with chronic inflammation, modulation of the immune system in the host, change in glucose metabolism, destabilization of suppressor tumor proteins, stimulation of angiogenesis, activation of invasion and metastasis [35].

Thus, the present study experimentally evaluated the effect of infection induced by *Plasmodium berghei* ANKA on the nociceptive and inflammatory responses evoked by the presence of a solid Ehrlich tumor in mice.

## 2. Material and Methods

### 2.1. Animal

The study was conducted using male BALB/c mice (20–25 g) from the central animal house at Fiocruz Rondônia. Each mouse was randomly assigned to an experimental group composed of 6-7 mice per group. Each group was housed in a single cage with free access to food and water and maintained at a controlled temperature (23 ± 1°C) in a 12 h light/dark cycle. The experiments were approved by the Commission of Ethics in Animal Research of the Fiocruz Rondônia (protocol number 2016/07 and 2017/02).

### 2.2. Malaria Infection

The model of noncomplicated malaria was established with a chloroquine-sensitive *Plasmodium berghei* ANKA strain provided by the Bioassay Platform for Malaria and Leishmaniasis (Fiocruz RO, Brazil). BALB/c mice were administered intraperitoneally with 0.2 ml of a solution containing 10^7^ red cells parasitized by the *Plasmodium berghei* ANKA strain. BALB/c mice were chosen because they were resistant to developing complicated malaria [36, 37]. In the control group, the animals were treated with RPMI-1640 medium following the same protocol for malaria infection. The development of infection was determined by the analysis of blood parasitemia.

### 2.3. Parasitemia

The blood parasitemia of the *Plasmodium berghei* ANKA strain was determined by counting parasitized red cells in blood smears stained with a Panoptical Fast Stain Kit (Giemsa-based stain), according to de Oca et al. [38]. Blood smears were prepared with 1-2 drops (~50 *μ*l) of whole blood obtained by tail incision with scissors. After complete drying, the blood smears were examined under a light microscope using a 100x oil immersion objective. The parasitized red cells were obtained by counting about 1000 red cells, which were presented by the percentage of parasitized red cells. Negative parasitemia was defined when the parasites did not observe each of the 100 visualized fields.

Parasitemia was determined on the third and tenth days after infection. The colored micrographs were obtained from a representative parasitemia slide of each experimental group using a light microscope (NIKON Ni-E) coupled with a CMOS image sensor. The micrographs received linear adjustments in brightness and contrast in the whole image using Photoshop (version 23.5.0).

### 2.4. Antimalarial Treatment

Antimalarial treatment was made with chloroquine. Chloroquine and its dose regimen were based on the World Health Organization (WHO) recommendation to treat noncomplicated malaria infection nonresistant to chloroquine [39] and the official guidelines of the Brazilian Ministry of Health to treat noncomplicated malaria [40]. Chloroquine was diluted in phosphate-buffered saline (PBS, pH 7.4) and administered orally for three consecutive days [41], from the seventh day to the ninth day after infection. The doses of chloroquine were 8.6 mg/kg (on the first day of treatment) and 6.45 mg/kg (on the second or third days), according to the protocol recommended for adult humans with 70 kg body mass. Mice in the control group were treated with phosphate-buffered saline (PBS).

### 2.5. Preparation and Inoculation of Ehrlich Tumor Cells

Swiss Webster female mice (25-30 g) were initially used as Ehrlich tumor cells. Cryopreserved Ehrlich tumor cells were thawed and inoculated intraperitoneally in Swiss Webster mice before being used in experimental animals, according to Calixto-Campos et al. [13]. After ten days, Ehrlich tumor cell ascitic fluid was collected by peritoneal cavity puncture. The ascitic fluid was washed in PBS (pH 7.4), centrifuged (200 g/*f* for 10 min), and again washed three times with PBS. The viability of tumor cells was determined by the 0.5% trypan blue exclusion assay in a Neubauer chamber.

After determining its concentration, the solution containing Ehrlich tumor cells was resuspended at a final concentration of 1 × 10^6^ in 25 *μ*l of PBS. Finally, the mouse was subcutaneously injected into the right hind paw with 25 *μ*l of suspension containing 1 × 10^6^ Ehrlich tumor. The control group was formed for mice injected with 25 *μ*l of PBS.

### 2.6. Evaluation of Paw Edema Induced by Ehrlich Tumor

The volume of the paw was measured using a paw plethysmometer, as described by Morris [42]. The hind paw was immersed in the container for evaluation, and the volume of liquid dislocated (in milliliters) was recorded. Data are represented with the delta percentage (*Δ*%) of variation in paw volume between the hind paws. The *Δ*% was calculated using the formula Δ% = [(CP/TP) − 1]∗100, in which CP and TP represent, respectively, the volume of the right hind paw (control paw not inoculated with tumor cells; CP) and the left hind paw (paw inoculated with tumor cells; TP).

### 2.7. Assessment of Mechanical Nociceptive Response

The mechanical nociceptive response was evaluated by measuring the frequency of withdrawal of the paw in ten applications of the von Frey filament, according to Nascimento Jr et al. [43]. Initially, the mice were placed in individual acrylic boxes with a metal mesh floor, where they remained for habituation. A mirror was placed above the animals to visualize the plantar region of their hind paws. The von Frey filament of 4 g/f (39 mN/f) was applied to the central region of the right hind paw of the plantar surface with the necessary pressure to cause the filament to bend. After applying the von Frey filament, withdrawal from the hind paw, lick or shake was considered a positive nociceptive response.

### 2.8. Assessment of Thermal Nociceptive Response

The hot plate test was used with the experimental model of the thermal nociceptive response (thermal hyperalgesia), as previously described by Eddy and Leimbach [44]. This test evaluates the time (threshold in seconds) for experimental animals to exhibit the behavior of licking or shaking the paws in response to exposure of the paws to a plate (Hot Plate HP-2002, Insight Equipamentos, Ribeirão Preto, SP, Brazil) automatically heated at 55°C. The cut-off time was set at 20 seconds to avoid tissue damage. Three consecutive measurements were obtained with intervals of five minutes between them using the average times for statistical evaluation.

### 2.9. IL-1*β* and TNF-*α* Dosage

After the conclusion of the experimental tests, peripheral blood was collected from mice through the retroorbital venous plexus, centrifuged, and serum separated and stored in a freezer at -80°C. Cytokine analysis (TNF-*α* and IL-1*β*) was performed using an enzyme-linked immunosorbent assay (ELISA) according to the manufacturer's recommendations (Kit BD Mouse). The colorimetric reaction was read in a microplate reader (ASYS UVM 340) at 450 nm.

### 2.10. Method of Euthanasia

At the end of peripheral blood collection, mice were sacrificed with an anesthetic overdose induced by an intraperitoneal injection of ketamine (180 mg/kg) + xylazine (24 mg/kg), followed by cervical dislocation.

### 2.11. Experimental Design

**Figure 1:**
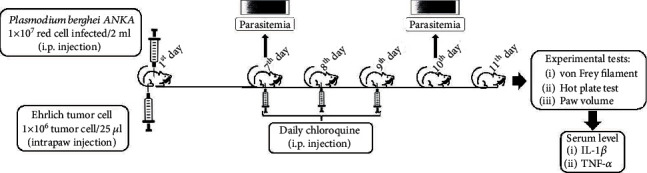
Experimental design of the study. First day: the intraperitoneal injection of red cells infected with *Plasmodium berghei* ANKA and intraplantar injection of Ehrlich tumor. Seventh to ninth day: daily oral treatment with chloroquine. Seventh and tenth day: parasitemia blood. Eleventh day: experimental tests and peripheral blood collection for dosing IL-1*β* and TNF-*α*.

On the first day of the experiment, the mice were infected with *Plasmodium berghei* and paw-injected with Ehrlich tumor cells. On the 7^th^ experimental day, the mice were treated daily with chloroquine for three days. On the 11^th^ experimental day, the mice were evaluated on the von Frey filament test, the hot plate test, and the paw volume test. Parasitemia was evaluated on the 7^th^ and 10^th^ experimental days. In the final phase of the experiments, peripheral blood was collected to determine the serum level of cytokines. The experimental design of the study is represented in Figure 1.

### 2.12. Statistical Analysis

Data were represented as mean ± SEM (standard error of the mean) or median with a 95% CI (confidence interval). The normal distribution was analyzed using the Shapiro-Wilk test. The influence of the different treatments on the biological responses to data with a normal distribution was analyzed using univariate analysis of variance (one-way analysis of variance) followed by Tukey's post hoc test. All statistical analyses were performed using the GraphPad Prism 9 statistical software, and the significance level was set at 0.05. The statistical analysis data are presented in Table 1 (supplementary data).

## 3. Results

**Figure 2:**
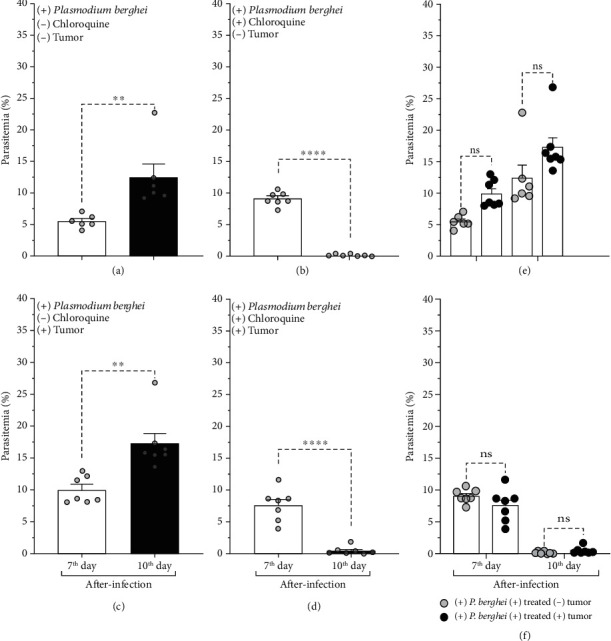
Blood parasitemia in mice infected with *Plasmodium berghei* ANKA on the seventh and tenth days after infection. Parasitemia was evaluated in mice treated or not treated with chloroquine and inoculated or not inoculated with Ehrlich tumor in the right hind paw. (a, c) Mice treated with vehicle. (b, d) Chloroquine-treated mice. (a, b) Mice inoculated with the vehicle in the hind paw. (c, d) Mice inoculated with Ehrlich tumor in the hind paw. Blood parasitemia in the presence of Ehrlich tumor in mice (e) not treated with or (f) treated with chloroquine. The columns represent the mean ± standard error of 6-7 mice. Cycles represent each mouse in the group. ^∗^Difference statistically significant. ^∗∗^*p* ≥ 0.0018. ^∗∗∗∗^*p* < 0.0001. ns = difference statistically nonsignificant.

The present study evaluated the manifestation of the nociceptive and inflammatory responses induced by solid Ehrlich tumor cells in the presence of infection with *Plasmodium berghei* in mice treated and untreated with antimalarials. Initially, we confirmed the development of *Plasmodium berghei* infection by identifying the forms of ring trophozoite in the blood smear on the seventh and 10^th^ days after infection. Parasitemia on the 10^th^ day after infection was significantly higher than on the 7^th^ day (Figures 2(a) and 2(c)). The progression of *Plasmodium berghei* was not altered by the presence of the Ehrlich tumor (Figures 2(c) and 2(e)). Daily chloroquine treatment significantly reduced parasitemia (Figures 2(b) and 2(d)). The suppressive effect of chloroquine on parasitemia did not change in the presence of the Ehrlich tumor (Figures 2(d) and 2(f)).

**Figure 3:**
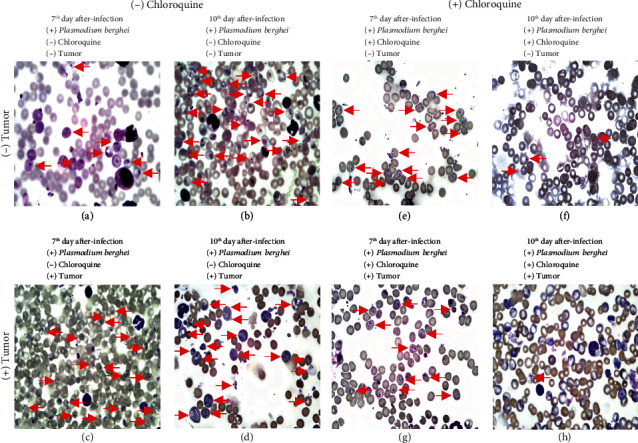
Photomicrography obtained from blood smear on days 7 and 10 after *Plasmodium berghei* ANKA infection. In (a)–(d), mice were not treated with chloroquine. In (e)–(h), the mice were treated with chloroquine. In (c) and (d) and (g) and (h), mice were inoculated with the Ehrlich tumor. Red arrows indicate ring-form trophozoites. Photomicrography was obtained for an optic microscope (NIKON Ni-E) at 100x magnification.

The progression of *Plasmodium berghei* infection and the effectiveness of chloroquine treatment were additionally presented in photomicrographs obtained from blood smears. Photomicrography was representative of the data shown in parasitemia (Figure 2) and reinforced the diversity of ring-form trophozoites in red cells on the seventh and tenth days after infection (Figure 3) in the different groups evaluated in the study.

**Figure 4:**
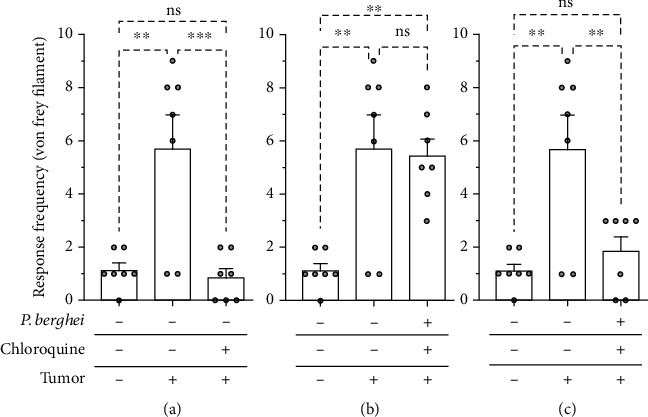
Mechanical hyperalgesia induced by Ehrlich tumor in mice infected with *Plasmodium berghei* and treated with chloroquine. (a) Mice with solid Ehrlich and treated with chloroquine. (b) Mice with solid Ehrlich tumor and infected with *Plasmodium berghei*. (c) Mice with solid Ehrlich tumor, treated with chloroquine, and healed from *Plasmodium berghei* infection. The columns represent the mean ± standard error of 6-7 mice. The cycles represent each mouse for the group. ^∗^Difference statistically significant. ^∗∗^*p* ≥ 0.0015. ^∗∗∗^*p* = 0.0009. ns = difference statistically nonsignificant.

The experimental groups were evaluated in models of mechanical and thermal pain and inflammation. The Ehrlich tumor injected into the hind paw increased mechanical nociceptive in response to the application of the von Frey filament compared to the control group (Figure 4). Mice treated with chloroquine had a reduction in tumor-induced mechanical nociception (Figure 4(a)). The mechanical nociception induced by the tumor did not change in mice infected with *Plasmodium berghei* (Figure 4(b)). Mice treated with chloroquine and healed from malaria infection had a significant reduction in mechanical nociception induced by the solid Ehrlich tumor (Figure 4(c)). Therefore, chloroquine treatment presents a mechanical antinociceptive effect in mice with solid Ehrlich tumor. The mechanical nociceptive response induced by the tumor did not change in mice infected with *Plasmodium berghei*. However, the mechanical nociceptive response induced by the tumor was significantly attenuated in chloroquine-treated mice and healed from malaria infection.

**Figure 5:**
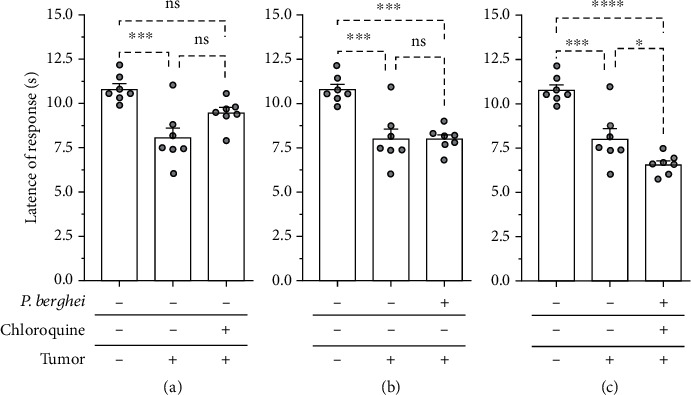
Thermal hyperalgesia induced by intraplantar inoculation of Ehrlich tumor in mice infected with *Plasmodium berghei* and treated with chloroquine. (a) Mice with solid Ehrlich and treated with chloroquine. (b) Mice with solid Ehrlich tumor and infected with *Plasmodium berghei*. (c) Mice with solid Ehrlich tumor, treated with chloroquine, and healed from *Plasmodium berghei* infection. Columns represent the mean ± standard error of 6-7 mice. The cycles represent each mouse for the group. ^∗^Difference statistically significant. ^∗^*p* < 0.05. ^∗∗∗^*p* ≥ 0.0003. ^∗∗∗∗^*p* < 0.0001. ns = difference statistically nonsignificant.

In the presence of the Ehrlich tumor, the thermal nociceptive threshold was significantly reduced compared to the control group, indicative of thermal hyperalgesia (Figures 5(a)–5(c)). Thermal hyperalgesia induced by Ehrlich tumor was not changed in chloroquine-treated mice (Figure 5(a)). *Plasmodium berghei* infection did not change the thermal nociception induced by the Ehrlich tumor (Figure 5(b)). Mice treated with chloroquine and healed from malaria infection had a significant reduction in thermic nociception threshold induced by the solid Ehrlich tumor (Figure 5(c)). Therefore, isolated chloroquine treatment or malaria infection did not change the thermal hyperalgesia induced by the tumor. However, tumor-induced thermal hyperalgesia was significantly increased in chloroquine-treated mice and healed from malaria infection.

**Figure 6:**
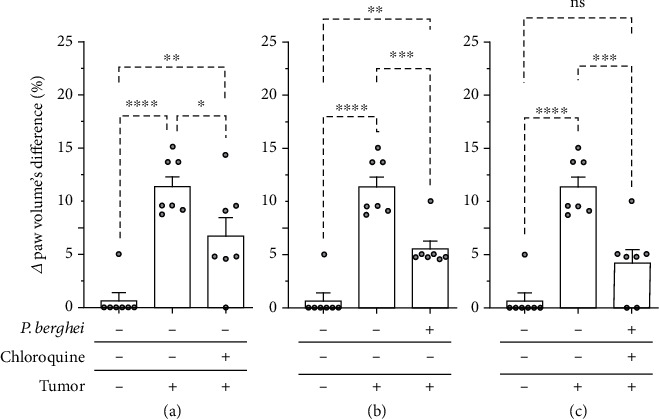
Paw edema induced by intraplantar inoculation of Ehrlich tumor in mice infected with *Plasmodium berghei* and treated with chloroquine. (a) Mice with solid Ehrlich and treated with chloroquine. (b) Mice with solid Ehrlich tumor and infected with *Plasmodium berghei*. (c) Mice with solid Ehrlich tumor, treated with chloroquine, and healed from *Plasmodium berghei* infection. The columns represent the mean ± standard error of 6-7 mice. The cycles represent each mouse in the group. ^∗^Difference statistically significant. ^∗^*p* < 0.05. ^∗∗^*p* ≥ 0.0018. ^∗∗∗^*p* = 0.0003. ^∗∗∗∗^*p* < 0.0001. ns = difference statistically nonsignificant.

We also showed that inoculation of the Ehrlich tumor increased the volume of the paw compared to the noninoculated paw (Figures 6(b)–6(d)), indicating the development of paw edema. Ehrlich tumor-induced paw edema was significantly inhibited in animals treated with chloroquine (Figure 6(a)) or infected with *Plasmodium berghei* (Figure 6(b)). Paw edema was also significantly inhibited in chloroquine-treated mice and healed from malaria infection (Figure 6(c)). These results indicate the antiedematogenic effect of chloroquine and the infection of *Plasmodium berghei* in mice with solid Ehrlich tumor. Furthermore, the antiedematogenic effect was also observed in mice healed of malaria infection after chloroquine treatment.

**Figure 7:**
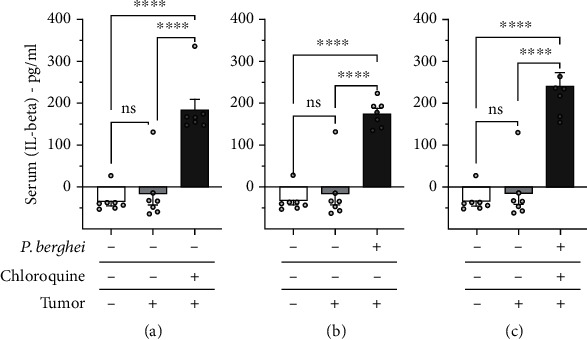
Serum level of IL-1*β* in mice inoculated with Ehrlich tumor, infected with *Plasmodium berghei*, and treated with chloroquine. (a) Mice noninoculated with Ehrlich tumor, noninfected with *Plasmodium berghei*, and treated with chloroquine. (b) Mice inoculated with Ehrlich tumor, infected with *Plasmodium berghei*, and nontreated with chloroquine. (c) Inoculated with Ehrlich tumor, infected with *Plasmodium berghei*, and treated with chloroquine. Columns represent the mean ± standard error of 6-7 mice. Cycles represent each mouse for the group. ^∗∗∗∗^*p* < 0.0001. ns = difference statistically nonsignificant.

**Figure 8:**
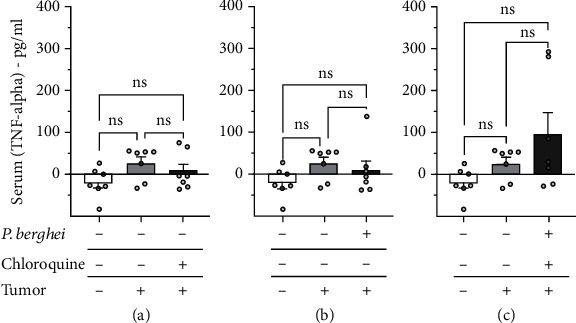
Serum level of TNF-*α* in mice inoculated with Ehrlich tumor, infected with *Plasmodium berghei*, and treated with chloroquine. (a) Mice noninoculated with Ehrlich tumor, noninfected with *Plasmodium berghei*, and treated with chloroquine. (b) Mice inoculated with Ehrlich tumor, infected with *Plasmodium berghei*, and nontreated with chloroquine. (c) Inoculated with Ehrlich tumor, infected with *Plasmodium berghei*, and treated with chloroquine. Columns represent the mean ± standard error of 6-7 mice. Cycles represent each mouse for the group. ns = difference statistically nonsignificant.

The serum level of the cytokines IL-1*β* and TNF-*α* was determined in mice injected with the solid Ehrlich tumor. The results show that the Ehrlich tumor did not induce an increase in serum levels of IL-1*β* (Figure 7) and TNF-*α* (Figure 8) compared to the control group. The serum level of IL-1*β* (Figure 7(a)), but not TNF-*α*, increased in mice with the solid Ehrlich tumor and treated with chloroquine. Mice injected with the solid Ehrlich tumor and infected with *Plasmodium berghei* presented an elevation of serum level of IL-1*β* (Figure 7(b)), but not TNF-*α* (Figure 8(b)). Mice with the solid Ehrlich tumor that was treated with chloroquine and healed from malaria infection presented an elevation of serum level of IL-1*β* (Figure 7(c)), but not TNF-*α* (Figure 8(c)), compared to the control.

## 4. Discussion

In the present study, we create an experimental condition of coexistence of these diseases to analyze the influence of malaria infection on the development of mechanical and thermal hyperalgesia and inflammation induced by Ehrlich tumor cells subcutaneously inoculated in the hind paw of the mouse. Furthermore, we evaluated the latte effect of malaria treated and healed on hyperalgesia and inflammation induced by the Ehrlich tumor. We used the Ehrlich solid tumor as an animal model of carcinoma and *Plasmodium berghei* as a model of uncomplicated malaria, which develops pathophysiological characteristics in humans.

Studies show that malaria and cancer can mutually influence some of their pathophysiological manifestations [30]. Our results show for the first time that the nociceptive response or the local inflammatory induced by the Ehrlich solid tumor changes during malaria infection and persists after treatment and heals the malaria infection. On the other hand, we cannot see any change in the blood development of malaria infection in the presence of an Ehrlich solid tumor, as verified by blood parasitemia.

More complex changes were observed in mice treated and healed of malaria. These mice had a significant attenuation of mechanical nociception and paw edema induced by the Ehrlich solid tumor. On the contrary, the thermal nociception induced by the Ehrlich tumor increased in these mice. Clinical evidence shows that patients who heal from malaria (aparasitemic) after effective antimalarial treatment can develop late neurological disorders that can occur anywhere from 0 to 60 days after parasitemia clearance [45]. Confusion, convulsion, ataxia, headache, abdominal pain, weakness, somnolence, and cognitive deficit are some neurological abnormalities observed in these patients [45, 46]. This neurological manifestation is related to abnormalities in the subcortical areas, brainstem, thalamus, and cerebellum observed by magnetic resonance imaging [46].

The use of antimalarials, such as mefloquine and chloroquine, for prophylaxis or the treatment of malaria is also associated with the development of neurological disorders [47, 48]. Treatment of malaria with chloroquine can produce a neurotoxic effect and a risk of neurological disorders, including seizures, psychotic episodes, involuntary movements, and extrapyramidal symptoms [47]. Studies suggest a synergistic interaction between antimalarial and malaria infection in developing neurological abnormalities [45]. In this sense, the neurologic abnormality resulting from chloroquine associated with malaria treatment could influence the nociceptive response induced by the solid Ehrlich tumor. Furthermore, these unprecedented results show that the association of malaria with chloroquine influences the tumor-induced nociceptive response based on the applied modality of the nociceptive stimulus.

Malaria infection in mice not treated with chloroquine inhibited paw edema without changes in the mechanical and thermal nociception induced by the Ehrlich solid tumor. The inhibitory effect of malaria on edema and Ehrlich tumor growth may be related to its ability to activate the immune response by inhibiting tumor angiogenesis and neutralizing the immunosuppressive microenvironment [24, 49]. Malaria is recognized by producing pain symptoms in humans [50, 51] or analgesia in the experimental model of inflammatory pain in mice infected with *Plasmodium berghei* [23]. These studies showing malaria-induced pain or analgesia were evaluated without comorbidity with chronic diseases or after clinical cure of malaria infection. In this sense, the inability of malaria to change the nociceptive response can be associated with the inherent characteristic of chronic pain induced by solid cancer.

Treatment with chloroquine in mice not infected with *Plasmodium berghei* also changed the development of edema and nociception induced by the Ehrlich tumor. The inhibitory effect of chloroquine on developing Ehrlich tumor-induced paw edema may be related to growth inhibition, inhibition of autophagy, and induction of cancer cell apoptosis [52, 53]. Chloroquine significantly attenuated mechanical and thermal nociception and edema induced by the Ehrlich tumor. Tsagareli et al. [54] showed in mice the development of a mechanical and thermal nociceptive response induced by intraplantar injection of chloroquine, which is inhibited by the transient receptor potential antagonist ankyrin 1 (TRPA1). Furthermore, chloroquine-induced nociception may be related to its cytotoxic effects due to its lysosomotropic and lysosomal acidification properties, leading to neurotoxicity [55–57]. The antinociceptive effect of chloroquine in the presence of an Ehrlich tumor may be due to its antitumor properties, especially by inhibiting tumor growth [52, 58]. Furthermore, these results may arise from chloroquine's selective interaction with different populations of neurons involved in the nociceptive response [54, 59, 60].

In our current investigation, we assessed the levels of IL-1*β* and TNF-*α*, two key players in the pathophysiologic aspects of cancer and malaria, particularly inflammation and pain. Our findings revealed a significant elevation in IL-1*β*, but not TNF-*α*, in mice with tumors and treated with chloroquine, in mice infected with *Plasmodium berghei*, and in mice with tumors and healed of malaria. Interestingly, tumor or malaria infection did not significantly alter the serum level of IL-1*β* and TNF-*α*.

IL-1*β* and TNF-*α* are pyrogens cyclically released by monocytes, macrophages, and neutrophils that participate in universal mechanisms of systemic inflammation and febrile response in infectious and noninfectious diseases, such as malaria and cancer [20, 61, 62]. In malaria, macrophages release IL-1*β* and TNF-*α* after recognizing PAMPs such as glycosylphosphatidylinositol and hemozoin [20]. Increased levels of IL-1*β* and TNF, beyond TGF-b and IL-10, are associated with the major severity of malaria [22, 63, 64]. Some clinical manifestations of malaria, such as fever, rigors, chills, fatigue, headache, thrombocytopenia, hypotension, anorexia, vomiting, nausea, and diarrhea, can be mimicked with an infusion of recombinant IL-1 and TNF-*α* [19, 65].

The antimalarial chloroquine, a lysosomotropic drug, exhibits a dual effect on the proinflammatory cytokine release. In leukocytes, chloroquine demonstrates a dose-dependent decrease in TNF-*α* and IL-1 secretion induced by lipopolysaccharide [66]. In sterile conditions, chloroquine inhibits autophagy, potentiates the action of IL-1*β*, and reduces IL-1 receptor internalization and degradation in macrophages [67]. This evidence suggests that the effect of chloroquine in increasing the serum level of IL-1*β* could be associated with the inhibition of autophagy of cytokines for inflammatory cell activation in response to the presence of a tumor.

IL-1*β* and TNF-*α* exhibit a dual effect, demonstrating protective effects during the innate immune response against pathogens and tumors and presenting harmful effects during cancer development. In several solid tumors, the production of IL-1*β* is upregulated and is associated with cachexia, invasion, angiogenesis, and metastasis [68–70]. In the tumor microenvironment, IL-1 promotes an immunosuppressive effect in antitumor cells such as activated macrophages (M2), tumor-associated neutrophils, regulatory B cells, and T helper 17 [71, 72]. Conversely, IL-1 is also associated with protective acts against tumors, exhibiting antitumor activity associated with the regression of several tumors.

The increased serum concentration of IL-1*β* was also related to mechanical antinociception and thermal hyperalgesia. Intraperitoneal injection of IL-1*β* induces thermal hyperalgesia, and this effect is inhibited by intracerebroventricular injection of nonsteroidal anti-inflammatory drugs [73, 74]. Moreover, evidence indicates that peripheral IL-1*β* can change brain function related to nociceptive behaviors [75]. Thus, intrathecal injection of IL-1*β* attenuates the carrageenan-induced hyperalgesia [76] and enhances the nociceptive threshold to mechanical stimuli [77].

Thus, our results show that the increase in serum concentration of IL-1*β* is related to the inhibition of tumor growth/edema in the paws of mice. An increased serum concentration of IL-1*β* in the presence of chloroquine and malaria could neutralize the immunosuppressive microenvironment of the solid Ehrlich tumor. Furthermore, the increased serum concentration of IL-1*β* could also be a contributing factor to the mechanical antinociception and thermal hyperalgesia observed in mice with tumors and healed of malaria. Moreover, its dual action in the brain and its peripheral effect could potentially explain the opposite effect of chloroquine in mechanical and thermal nociception.

## 5. Conclusion

Finally, the results show for the first time how the nociceptive and inflammatory manifestations induced by the solid Ehrlich tumor manifest themselves in animals infected with *Plasmodium berghei* ANKA and treated with antimalarial chloroquine. The most complex results were observed in mice treated with chloroquine and healed of malaria. Although there are differences in nociceptive and inflammatory manifestations in animal models of malaria compared to the classic manifestations of malaria in humans, the study draws attention to the complexity of some clinical manifestations that can develop in individuals with malaria treated with antimalarials and who have had cancer concurrently. In developing countries, malaria and antimalarial therapy can coexist with cancer, bringing about beneficial clinical responses, while they can worsen others in cancer patients. Therefore, human observational studies are needed to assess how the interaction of different types of malaria and antimalarial treatments can influence the development of coexisting tumors.

## Data Availability

The data supporting this study's findings are available from the corresponding author, Quintino Moura Dias, upon reasonable request.

## Supplementary Materials

Supplementary Materials The statistical analysis data of Figures 2 and 4–8 is presented in the supplementary file.
