# Supplementary material for: The Nociceptive and Inflammatory Responses Induced by the Ehrlich Solid Tumor Are Changed in Mice Healed of Plasmodium berghei Strain ANKA Infection after Chloroquine Treatment
Source: J Parasitol Res. 2024 May 14;2024:3771926. doi: 10.1155/2024/3771926 (PMC11108701; doi:10.1155/2024/3771926)
Supplement: Supplementary Materials — The statistical analysis data of Figures 2 and 4–8 is presented in the supplementary file. [file 3771926.f1.docx]

Supplementary data

TABLE 1: Statistical analysis data of experimental results

Figure 2

| Unpaired t test | | | | |
| --- | --- | --- | --- | --- |
|  | **FIGURE 2A** | **FIGURA 2B** | **FIGURE 2C** | **FIGURE 2D** |
| **P value** | 0,0086 | <0,0001 | <0,0001 | <0,0001 |
| **t; df** | 3,261; 10 | t=21,69, df=12 | t=21,69, df=12 | t=7,236, df=12 |

| One-way ANOVA | | |
| --- | --- | --- |
|  | **FIGURE 2E** | **FIGURE 2F** |
| **P value** | P<0,0001 | P<0,0001 |
| **F (DFn, DFd)** | F (3, 22) = 12,36 | F (3, 24) = 76,39 |

Figure 4

| One-way ANOVA | | | |
| --- | --- | --- | --- |
|  | **FIGURE 4A** | **FIGURE 4B** | **FIGURE 4C** |
| **P value** | P=0,0004 | P=0,0016 | P=0,0018 |
| **F (DFn, DFd)** | F (2, 18) = 12,46 | F (2, 18) = 9,390 | F (2, 18) = 9,165 |

Figure 5

| One-way ANOVA | | | |
| --- | --- | --- | --- |
|  | **FIGURE 5A** | **FIGURE 5B** | **FIGURE 5C** |
| **P value** | P=0,0007 | P=0,0001 | P<0,0001 |
| **F (DFn, DFd)** | F (2, 18) = 11,27 | F (2, 18) = 16,03 | F (2, 18) = 29,44 |

Figure 6

| One-way ANOVA | | | |
| --- | --- | --- | --- |
|  | **FIGURE 6A** | **FIGURE 6B** | **FIGURE 6C** |
| **P value** | P<0,0001 | P<0,0001 | P<0,0001 |
| **F (DFn, DFd)** | F (2, 18) = 18,46 | F (2, 18) = 40,53 | F (2, 18) = 27,18 |

Figure 7

|  | **FIGURE 7A** | **FIGURE 7B** | **FIGURE 7C** |
| --- | --- | --- | --- |
| **P value** | P<0,0001 | P<0,0001 | P<0,0001 |
| **F (DFn, DFd)** | F (2, 18) = 31,71 | F (2, 18) = 45,00 | F (2, 18) = 39,46 |

Figure 8

|  | **FIGURE 8A** | **FIGURE 8B** | **FIGURE 8C** |
| --- | --- | --- | --- |
| **P value** | P=0,1256 | P=0,2030 | P=0,0663 |
| **F (DFn, DFd)** | F (2, 18) = 2,334 | F (2, 18) = 1,744 | F (2, 18) = 3,167 |
